# *Hericium erinaceus* (Bull.) Pers. Ethanolic Extract with Antioxidant Properties on Scopolamine-Induced Memory Deficits in a Zebrafish Model of Cognitive Impairment

**DOI:** 10.3390/jof7060477

**Published:** 2021-06-12

**Authors:** Mihai-Vlad Valu, Liliana Cristina Soare, Catalin Ducu, Sorin Moga, Denis Negrea, Emanuel Vamanu, Tudor-Adrian Balseanu, Simone Carradori, Lucian Hritcu, Razvan Stefan Boiangiu

**Affiliations:** 1 Department of Natural Sciences, Faculty of Science, University of Pitesti, Targu din Vale Street, 110040 Pitesti, Romania; mihai.valu@upit.ro (M.-V.V.); cristina.soare@upit.ro (L.C.S.); 2 Regional Center of Research & Development for Materials, Processes and Innovative Products Dedicated to the Automotive Industry, University of Pitesti, 11 Doaga Street, Arges, 110440 Pitesti, Romania; catalin.ducu@upit.ro (C.D.); sorin.moga@upit.ro (S.M.); den-is.negrea@upit.ro (D.N.); 3 Faculty of Biotechnology, University of Agronomic Science and Veterinary Medicine, 59 Marasti Blvd, 1 District, 011464 Bucharest, Romania; email@emanuelvamanu.ro; 4 Experimental Research Center for Normal and Pathological Aging, University of Medicine and Pharmacy of Craiova, 2 Petru Rareş Street, 200349 Craiova, Romania; adrian.balseanu@umfcv.ro; 5 Department of Pharmacy, “G. d’Annunzio” University of Chieti-Pescara, Via dei Vestini 31, 66100 Chieti, Italy; simone.carradori@unich.it; 6 Department of Biology, Alexandru Ioan Cuza University of Iasi, Bd. Carol I, No. 11, 700506 Iasi, Romania; razvan.boiangiu@student.uaic.ro

**Keywords:** *Hericium erinaceus*, antioxidant activity, scopolamine, Alzheimer’s disease, zebrafish

## Abstract

*Hericium erinaceus* (*H. erinaceus*) is a rare and appreciated fungal species belonging to the division *Basidiomycota* used for centuries in traditional Chinese medicine for its medicinal value. This species of mushrooms brings the most diverse benefits for the human body, and can have beneficial effects for treating Alzheimer’s disease (AD). This study investigated whether ethanolic extract from the fungal biomass of *H. erinaceus* enhances cognitive function via the action on cholinergic neurons using the scopolamine (SCOP)-induced zebrafish (*Danio rerio*) model of memory impairment. The ethanolic extract from the fungal biomass of *H. erinaceus* was previously obtained using an ultrasonic extraction method (UE). The administration of *H. erinaceus* extract to zebrafish, with a pattern of AD induced by scopolamine, showed an improvement in memory evaluated by behavioral and biochemical tests on brain tissue. These results suggest that *H. erinaceus* has preventive and therapeutic potentials in managing memory deficits and brain oxidative stress in zebrafish with AD.

## 1. Introduction

**Figure 1:**
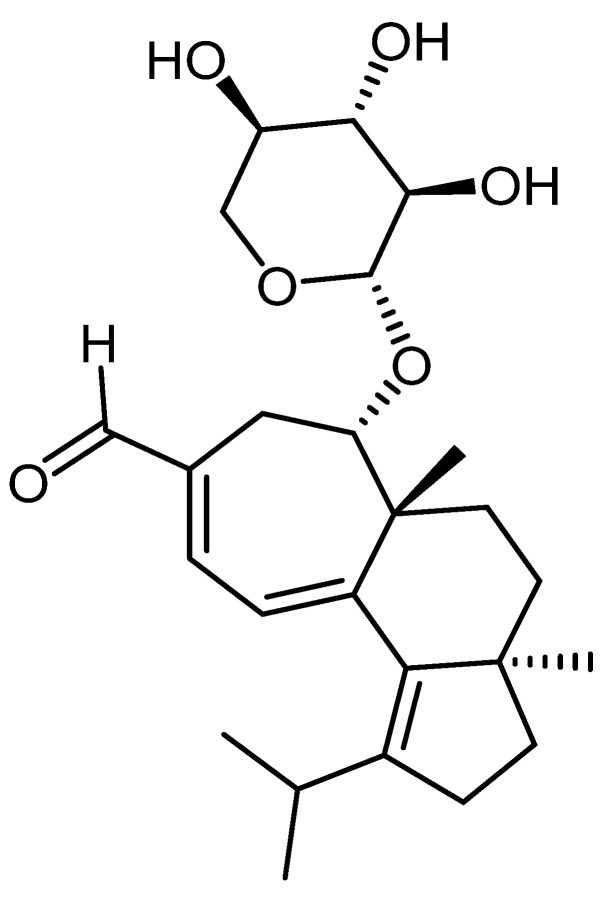
Chemical structure of erinacine A.

Alzheimer’s disease (AD) is a neurodegenerative condition that progressively destroys brain cells [1]. It is the most common form of dementia, affecting 60–65% of people with dementia [2]. AD is diagnosed in people over 65 years of age, but it can occur less often in much younger people [3]. Symptoms develop gradually and worsen over time, becoming severe enough to interfere with daily activities [4]. Current therapies for AD cannot stop the disease from progressing; they can temporarily slow the worsening of dementia symptoms and improve the quality of life of people with AD [5]. Global efforts are being made to discover ways to treat the disease through various herbs with medicinal effects [6], especially regarding medicinal mushrooms [7]. Mushrooms are a source of food that people have used since ancient times, being consumed in many parts of the world [8]. The exact number of mushroom species is still a matter of dispute for researchers, although about 200,000 species of fungi are known, of which only about 72,000 species are described (only 3000 are edible) [9]. Mushrooms are rich in vitamins, minerals, fiber, antioxidants, and water (80–90% content). Due to the many valuable substances they contain, mushrooms are used as natural medicine [10]. For example, the consumption of mushrooms is associated with a long and balanced life [11]. This therapy called mycotherapy aims to strengthen the entire body and restore the natural, healthy balance of all processes in the body. Mushrooms strengthen the immune system and activate the body’s self-healing capacity [9,10]. Mycotherapy is a prophylactic or accompanying treatment in conventional therapies, even severe diseases [12]. For example, the fungal species *Hericium erinaceus* (*H. erinaceus*) belongs, along with several other species, in the category of fungi with intense medical activity due to the biological compounds found in this species, such as polysaccharides (β-glucans) that have an immunostimulant effect, dietary fibers, unsaturated fatty acids, terpenes, peptides, glycoproteins, alcohols, tocopherols, and ascorbic acid endowed with anticancer and antimetastatic properties [13,14,15]. The genus *Hericium* produces phytochemical substances, erinacine and hericenone, which act on the brain-derived neurotrophic factor (BDNF) protein [16]. The diterpenoid erinacine A (Figure 1) was isolated from the fungal species *H. erinaceus* [17]. The compound erinacine A can stimulate nerve cell growth and regeneration and has led to research into its effects on the nervous system [18]. *H. erinaceus* extracts enhanced the nerve growth factor (NGF) mRNA and protein expression in the hippocampus, suggesting that the bioactive compounds in the extract could move across the blood-brain barrier (BBB), resulting in hippocampal neurogenesis [19]. Hericenones and erinacines can quickly move through the blood-brain barrier due to their small molecular sizes [20]. Most experiments have focused on these two main bioactive compounds [19,21]. Future tests evaluating erinacine A concentrations in the brain and blood may help explain these pathways in greater depth. Unfortunately, mycotherapy is still poorly understood and more studies and research are needed, based on which people can be informed so that they can use mushrooms, both in their diet and for treating diseases.

Both erinacine-based compounds and hericenone induce the biosynthesis of NGF in neurons [16]. In a study on rats, the administration of erinacine A led to an adjustment of NGFs in two areas of the brain, locus coeruleus and hippocampus [20,22,23,24,25,26]. These areas of the brain are usually affected in patients with dementia and AD. The administration of erinacine A in mice with Alzheimer’s and induced Parkinson’s improved the symptoms of both diseases [24,25,26]. A group of diterpenoids isolated from cultured mycelia of *H. erinaceus*, namely, erinacines, was demonstrated to be a potential enhancer of NGF biosynthesis in cultured astrocytes and increased production of NGF is correlated with proper neural growth and maintenance [22]. Notably, erinacine A has been reported to exhibit a protective effect against ischemic injury, Parkinson’s, and AD in vivo [20,24,25]. Therefore, erinacine A-enriched *H. erinaceus* attracts attention and may serve as a promising agent with neurotrophic activity, potentially easing neurodegenerative disorders [16]. Our research group has recently cultivated solid-state fungal biomass of *H. erinaceus* to obtain the biological compound erinacine A [27,28], validating it as the most optimal method of ultrasonic extraction (UE). Since the chemical synthesis of diterpenoids is a lengthy, multi-stage method with a low yield, it has been suggested that the biosynthesis of erinacine A in submerged culture reduces production costs [16,24]. Thus, in this article, studies are continued to validate the extract obtained by UE from the fungal biomass of *H. erinaceus* for antioxidant and neuroprotective effects. Simultaneously, the originality of the article results from the fact that this is the first study in which the neuroprotective effect of the ethanolic extract obtained from the fungal biomass of the species *H. erinaceus* by UE was studied on the zebrafish model. In this context, zebrafish small freshwater teleosts have become an attractive model for studies evaluating the activity of natural and synthetic compounds and their potential for therapies of various pathological conditions. [27]. As a result, this research investigated the neuroprotective properties of *H. erinaceus* extract obtained by UE, being a continuation of our group’s research on the antioxidant effect of this medicinal mushroom. AD hallmarks, represented by anxiety, depression, and short- and long-term memory impairment, were highlighted, attempting to observe significant structural modifications following the treatments administered, and improvement of physiological parameters.

## 2. Materials and Methods

### 2.1. Chemicals and Reagents

Extracts were obtained from the fungal biomass of *H. erinaceus* as previously reported [28]. Scopolamine, galantamine, imipramine, ethanol-ethyl acetate, and 2,4,6-*tris*(2-pyridyl)-1,3,5-triazine (TPTZ) were obtained from ThermoFisher Scientific (Darmstadt, Germany). The saline phosphate buffer, acetic acid, Tris hydrochloride, were all purchased from Sigma-Aldrich (Steinheim, Germany), and ethyl alcohol (96%) from Prodvinalco S.A. (Cluj-Napoca, Romania). Tetrazolium Blue Chloride, riboflavin, ethylenediaminetetraacetic acid (EDTA), sodium azide 0.1M solution (NaN_3_), glutathione reduced ethyl ester (GSH-MEE), trichloroacetic acid, 2,4-dinitrophenylhydrazine (DNPH), acetylthiocholine (ATC), 5,5′-dithio-bis-2-nitrobenzoic acid (DTNB) were purchased from Avantor^®^ company (Wien, Austria).

### 2.2. Extract from Fungal Material Preparation

The ethanolic extract from *H. erinaceus* (HE) was obtained according to the method previously described by our research group [29]. *H. erinaceus* fungal biomass was developed on a solid culture medium under controlled conditions. Finally, *H. erinaceus* biomass was subjected to the UE technique, using an ultrasonic processor (Hielscher UIP1000hdT, Berlin, Germany). The extraction conditions were 80% ethanol as the solvent, the extraction time was 45 min, and the solvent-material ratio 1:30 (g/mL). We used ultrasound to improve the efficiency of extraction. After the extraction, the samples were vacuum filtered and centrifuged for 5 min at 2500× *g*. Water and alcohol were removed from the supernatants using a rotary evaporator (Heidolph Hei-VAP Core, Schwabach, Germany). The last stage consisted of lyophilization of the samples (Christ α-1–2 LD plus, Osterode, Germany) to obtain a dry matter (D.M.), which was separated in quantities of 0.5, mg, 1 mg, and 3 mg and used in subsequent tests.

### 2.3. Animals and Drug Administration

The zebrafish, small tropical freshwater teleosts, has emerged as a prominent animal model for studying complex behaviors, including learning and memory [30]. In this study, 70 adult wild type, short-fin strain zebrafish were used. The female and male in a ratio of 50:50 were purchased from an authorized commercial dealer (Pet Product S.R.L., Bucharest, Romania) and were minimally 6 months of age (3  ±  0.5 cm and 0.4  ±  0.05 g). The number of animals (*n* = 10 per group) was chosen following biostatistical studies showing that a minimum number of animals are required. Animals were kept in 30-L housing tanks with unchlorinated water at a targeted temperature of 26 ± 2 °C and continuously aerated under 14:10 h light: dark photoperiod. The dechlorinated water was changed once every 3 days. The fish were acclimated to the laboratory environment for at least 14 days and were fed three times a day with commercial food NovoMalawi (JBL, Neuhofen, Germany). Drug doses, HE (0.5 mg/L, 1 mg/L, and 3 mg/L), and administration routes were chosen and adjusted based on previous studies demonstrating effects on memory in rodents [18,24,25,26]. Before performing a behavioral test, the fish were placed individually, for 1 h, in the scopolamine (SCOP 100 μM) solution for the experimental induction of the animal model of cognitive impairment. HE (0.5, 1, and 3 mg/L) was diluted with water and was administered to zebrafish by immersion for 1 h, once daily for 13 days, while SCOP (100 µM) was administered 30 min before each behavioral tests. The control group was immersed only in unchlorinated water. The SCOP solution was prepared before use and changed after each exposure to reduce the number of variables that may influence behavior. To evaluate the effects of the HE on memory processes and oxidative status in the experimental model, the subsequent batches of animals with mushroom treatment (0.5, 1, and 3 mg/L) were used. In advance, to determine the safety profile and toxicity assessment for doses of *H. erinaceus*, 60 zebrafish of both sexes were divided into four groups labeled as control and *H. erinaceus* (0.5, 1, and 3 mg/L). Any signs of toxicity and mortality were monitored for two weeks. Complex pharmacokinetics of *H. erinaceus* and other drugs have not yet been characterized in zebrafish. The behaviorally relevant dose ranges of *H. erinaceus* were determined by pilot studies, which tested the effects of concentrations ranging from ineffective to those having acutely toxic effects. The Faculty of Biology Ethics Committee for animal experimentation had previously accepted this report (No. 02/30.06.2020). It thoroughly complied with Directive 2010/63/EU of the European Parliament and of the Council of 22 September 2010, on the conservation of animals. The animals were treated with care, without causing them any discomfort or inconvenience, and every attempt was taken to mitigate cruelty and the number of animals used.

### 2.4. Behavioral Tasks for Cognitive Performance

Various behavioral tests are currently used to assess sensory functions—motor, social interactions, anxious and depressive behavior, multiple forms of cognitive function, or substance dependence [31]. Zebrafish was sometimes identified as an alternative model (relative to classic rodent models). Still, the term complementary model might be more appropriate since it addresses the use of fish in addition to traditional mammalian models [30]. In this study, anxiety manifestations of the zebrafish were assessed using a novel tank diving test (NTT), a Y-maze test (to assess novelty exploration), and a new object recognition test (NOR) for assessing short-term and long-term memory. The tests were performed successively with an interval of 2 days of rest between them and scheduled between 8:00 and 17:00. Animal behavior in cognitive tests was recorded by a video camera (Logitech C922 HD Pro Stream), and the films were analyzed using an ANY-maze, v6.1 (Stoelting Co., Woods-Dale, NY, USA). After performing the memory and anxiety tests, the zebrafish were euthanized. The whole brains were precisely excised and, with the rest of the body, were taken and used for the biochemical tests presented below.

#### 2.4.1. Novel Tank Diving Test (NTT)

In the new aquarium test, zebrafish show solid behavioral responses to novelty-induced anxiety [32,33]. This test was based on the animal’s instinct to seek protection in an unknown environment through diving, freezing, and reduced exploration [32,34]. As the fish gradually adapt to the new environment, there is usually an increase in the quest (e.g., increased locomotor activity, decreased immobility, and more entries in the upper half of the basin) [35]. These parameters (exploration, immobility, and upper half entries) can be applied to zebrafish models for anxiety, like the open field test (OFT) performed on rodents, in which mice or rats exhibit anxiety-like behavior [35]. The fish’s position in the aquarium (top or bottom) was considered an indicator of anxiety [36]. The fish will be transferred individually to a test aquarium and filmed for 6 min. The experimental apparatus consists of a trapezoidal glass aquarium with the following dimensions: 23.9 cm lower side × 28.9 cm upper side × 15.1 cm high and 15.9 cm diagonal. The upper width was 7.4 cm, and the lower was 6.1 cm; the aquarium was filled with 1.5-L water from the accommodation aquarium. A webcam was placed 40 cm from the test aquarium to ensure that the device was within the camera range and was used to monitor the location and swimming activity of the fish.

#### 2.4.2. Y-Maze Test

Before being placed in the behavioral equipment, the fish were given 5 min to rest in the water in the accommodation aquarium. The fish were tested in a glass aquarium in the shape of the letter Y with three arms 25 cm × 8 cm × 15 cm (length × width × height). Various geometric shapes (squares, triangles, and circles) made of white paper were visual cues (stimuli). They were placed on the outer walls of each arm, making them visible from inside the maze, and the remaining area was covered with black plastic self-adhesive foil. In the device, 3 L of the same water used in the accommodation aquarium was used to cover the visual cues. The maze arms were set randomly: the start arm from which the fish will start the test, the new arm that was locked during the initial test, but which was opened during the second test, and the permanently open arm. The central area was considered a neutral area and was therefore not considered in the analysis [37]. The Y-maze test consisted of two steps separated by an hour between them to assess the response to novelty (the fish placed in the device when the new arm was unlocked) and spatial recognition memory [37]. During the first stage (5 min of training), the fish could explore the start and open arm, but the new arm was kept closed. In the second stage (after one hour), the fish were placed in the start arm and allowed to access all three arms for 5 min. The time spent in the new arm was determined, along with parameters for locomotor activity (such as total distance traveled, average speed, rotation angle, and several lines crossed).

#### 2.4.3. Novel Object Recognition Test (NOR)

The object discrimination test, also known as the spontaneous object recognition test or the new object recognition test (NOR), is used to assess the ability to recognize a new object in the environment, which is one of the most popular paradigms used to evaluate memory [38]. The NOR test is beneficial for studying both short-term and long-term memory [39]. By simply manipulating the retention interval, defined as the period between the training session and the test session, it is possible to evaluate any type of memory [40]. The biggest advantage of this test is that it does not require external motivation, reward, or punishment, but habituation is necessary and can be completed in a relatively short period [38]. The NOR test has previously been studied in zebrafish, where the ability to discriminate between different objects and object recognition memory was assessed [38,39]. The temperature and pH of the water in the test aquarium were like that of the water in the housing aquarium. Because zebrafish express a three-dimensional swimming profile [41], we used a lower water level (the height of the water column was 6 cm) that allowed the fish to swim freely in horizontal directions. All experimental batches were subjected to the following steps: (1) habituation to the test aquarium (3 days); (2) the training session in which the animals were exposed to two identical objects; (3) induction of the model and exposure to treatment, and (4) in the test phase (1 h after the training phase), a novel object (N, green cube) replaced one of the copies of the familiar objects (F, red cubes), and the exploration time of each object was evaluated for 10 min. [42]. After training, the animals underwent a retention interval of one hour, during which they were given treatment. In the testing stage, the exploration time of each object (%) was evaluated. Preference percentages were calculated according to the following formula: (new object exploration time/(familiar object exploration time + new object exploration time)) × 100 [42,43]. The total exploration area, including the object (placed in the middle of the area), was 56.25 cm^2^ (7.5 cm × 7.5 cm) on each side of the aquarium (6.25% of the total area).

### 2.5. Biochemical Analysis

At the end of the behavioral tests, all zebrafish were euthanized by rapid cooling (10 min immersion in ice water, 2–4 °C). This is an easy, inexpensive method and does not cause biochemical or physiological changes that impede postmortem analysis. The technique required lowering the water temperature to 2–4 °C by adding ice (5 parts of ice and 1 part of water) and preventing the fish from touching the ice before becoming unconscious. The fish were taken from cold water and transferred to a dissection medium where they were dried by dabbing with a paper towel. The fish were decapitated, the heads were further subjected to precise dissection in the saline phosphate buffer, and their whole brains were isolated for a biochemical parameters assay. Since the anterodorsolateral pallium in the adult zebrafish brain shows high random neuronal activation embedded with rapid ripple oscillations (above 100 Hz), it seems to be the zebrafish equivalent of the mammalian hippocampus [44]. In this part of the study, we investigated whether the degradation of cognitive performance caused by the SCOP administration is associated with altered oxidative stress indices. Numerous experimental studies have shown that scopolamine causes oxidative stress, blocks the action of acetylcholine in the brain, and causes memory degradation [45,46]. The whole brain samples were weighed and homogenized. The homogenates obtained were centrifuged (15 min at 960× *g*), and the supernatant obtained was used to estimate the specific activities of acetylcholinesterase (AChE), superoxide dismutase (SOD), catalase (CAT), and glutathione peroxidase (GPX), along with glutathione (GSH), malondialdehyde (MDA), and carbonylated proteins levels.

#### 2.5.1. Acetylcholinesterase Activity

The acetylcholinesterase (AChE) activity was monitored from the brain samples using by the method of Ellman et al. [47] using acetylthiocholine (ATC) as an artificial substrate. The reaction mixture (600 µL final volume) contains 0.26 M phosphate buffer pH 7.4, 1 mM DTNB, and 5 mM ATC. The determination was performed by adding the supernatant to the reaction mixture to yellow (10 min at room temperature). The readings were taken using a spectrophotometer with a wavelength of 412 nm. Enzyme activity was expressed in nmol ACT/min/mg protein.

#### 2.5.2. Superoxide Dismutase

Superoxide dismutase activity (SOD, EC 1.15.1.1) was determined by monitoring its ability to inhibit the photochemical reduction of NBT (Nitroblue Tetrazolium Test). Each 1.5 mL reaction mixture contains 100 mM Tris/HCl (pH 7.8), 75 mM NBT, 2 μM riboflavin, 6 mM EDTA, and 200 μL supernatant. Monitoring the increase in absorption at 560 nm is followed by the production of formazan blue. One unit of SOD was defined as the amount needed to inhibit the rate of NBT by reducing it by 50% [48]. The enzyme activity was expressed in unit/mg protein.

#### 2.5.3. Catalase

The specific activity of catalase (CAT, EC 1.11.1.6) was determined based on the method described by Sinha [49]. The mixture reaction consists of 150 µL phosphate buffer (0.01 M, pH 7.0) and 100 µL supernatant. The reaction was triggered by the addition of 250 µL 0.16 M H_2_O_2_, incubated at 37 °C for 1 min, after which it was quenched by the addition of 1 mL of acetic acid. The reaction tubes were kept in a water bath for 15 min until the green appeared, and readings were taken on a spectrophotometer at 570 nm. The enzyme-free control tubes were stored under the same conditions. The activity of the enzyme was expressed in µmoles of H_2_O_2_ consumed/min/mg protein.

#### 2.5.4. Glutathione Peroxidase

A spectrophotometry test-analyzed glutathione peroxidase (GPX) activity was used. The mixture reaction consisted of 1 mL of 0.4 M phosphate buffer (pH 7.0) containing 0.4 mM EDTA, 1 mL of 5 mM NaN_3_, 1 mL of 4 mM GSH, and 200 µL of supernatant and was incubated at 37 °C for 5 min. Then, 1 mL of 4 mM H_2_O_2_ is added and incubated at 37 °C for 5 min. Excess GSH was quantified by the DTNB method described by Massarsky et al. [50]. A unit of GPX was defined as the amount of enzyme required to oxidize 1 nmol GSH/min. Enzyme activity was expressed in unit/mg protein.

#### 2.5.5. Glutathione

Generally, glutathione (GSH) is a tripeptide and is considered the most important form of storage and transport of low sulfur [51]. Glutathione is an essential component of the cellular antioxidant defense system and is directly involved in maintaining cellular redox homeostasis [52]. GSH was determined based on the method described by Fukuzawa et al. [53]. Thus, 200 μL of supernatant mixed with 1.1 mL of 0.25 M sodium phosphate buffer (pH 7.4) and 130 μL of 0.04% DNTB. Finally, the mixture was made up to 1.5 mL with distilled water, and the absorbance was read on a spectrophotometer at 412 nm. The total reduced GSH content was expressed in µg GSH/µg protein.

#### 2.5.6. Malondialdehyde

Malondialdehyde (MDA) is an indicator of lipid peroxidation and was measured spectrophotometrically using the thiobarbituric acid test [54]. 200 μL of supernatant mixed with 1 mL of 50% trichloroacetic acid in 0.1M HCl, were added to 1 mL of 26 mM thiobarbituric acid. After vortexing, the samples were kept at 95 °C for 20 min. Subsequently, the samples were centrifuged at 960× *g* for 10 min, and the supernatants were read at 532 nm. The calibration curve was then constructed, and the results were expressed in nmol/mg protein.

#### 2.5.7. Carbonylated Proteins

The degree of oxidation of hippocampal proteins was assessed by determining the level of carbonyl groups based on the methods described by other authors [55,56]. The supernatant was divided into two samples, each containing approximately 2 mg of protein. Protein precipitation occurred in both samples by adding 10% trichloroacetic acid (*w*/*v*, final concentration). One sample was treated with 2 N HCl, and the other sample with 0.2% (*w*/*v*) DNPH in 2 N HCl. Both samples were incubated at 25 °C and stirred at 5-minute intervals. The samples were precipitated again with 10% trichloroacetic acid (*w*/*v*, final concentration) and extracted with ethanol-ethyl acetate (1:1, *v*/*v*) and then precipitated with 10% trichloroacetic acid. They were subsequently dissolved in 8 M urea with 20 mM sodium phosphate buffer, pH 6.5. Insoluble cell debris was removed by centrifugation at 13,000× *g* at 4 °C. The absorbance was read on the spectrophotometer at 370 nm, comparing the samples treated with DNPH with HCl (control). The results are expressed in nmol DNPH/mg protein.

### 2.6. Statistical Analysis

Data obtained from animal experiments were expressed as mean, standard error (±S.E.M.). One-way analysis of variance (ANOVA) and Tukey’s post hoc multiple reference test evaluated statistical differences between the treatments and control, with treatment as a factor. One-way Kruskal-Wallis test with Dunn’s multiple comparison post hoc test was used for non-parametric data in the NOR test. The data were analyzed using GraphPad Prism 9.0 software. Pearson correlation coefficient (*r*) was evaluated to determine the correlation between behavioral scores, enzymatic activities, and lipid peroxidation.

## 3. Results

### 3.1. Effects of H. erinaceus Extracts on Anxiety in NTT Task

**Figure 2:**
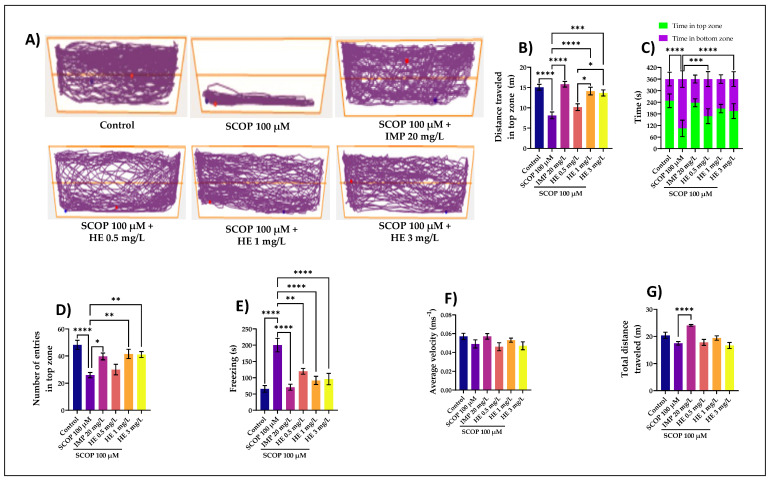
*Hericium erinaceus* ethanolic extract (HE: 0.5, 1, and 3 mg/L) improved the locomotion pattern and reduced anxiety in the novel tank-diving test (NTT). (**A**) Representative locomotion-tracking pattern of the control, scopolamine (SCOP: 100 µM), *Hericium erinaceus* (HE: 0.5, 1, and 3 mg/L) and imipramine (IMP: 20 mg/L) treated groups. (**B**) Representation of the distance traveled in the top zone in different groups. (**C**) The time spent by zebrafish in the top/bottom zone of the tank in different groups. (**D**) Representation of the number of entries in the top zone by zebrafish in the tank in different groups. (**E**) Representation of the freezing activity by zebrafish in the tank in different groups. (**F**) Representation of the average velocity of zebrafish in the tank in different groups. (**G**) Representation of the total distance traveled by zebrafish in the tank in different groups. Values are means ± S.E.M. (*n* = 10). For Tukey’s *post hoc* analysis: **** *p* < 0.0001, *** *p* < 0.001, ** *p* < 0.01, and * *p* < 0.05.

To minimize the novelty stress in the NTT task, we first investigated the zebrafish habituation response to the test apparatus. Zebrafish are transported individually from their home tank to the treatment in the novel tank with careful handling to reduce net stress. Starts were recorded and continued for 6 min. This test uses a vertical distribution in a novel environment as a validated behavioral test to assess anxiety-like behaviors in adult zebrafish [57,58]. When first introduced into a novel tank, zebrafish show a reproducible behavior of diving to the bottom and then, over time, they increase swimming to higher levels (Figure 2A). SCOP-treated groups exhibited a preference for the bottom zone, suggesting high levels of anxiety. The effects of the HE acute treatment on anxiety-like behavior in zebrafish exposed to SCOP (100 μM) were assessed in NTT by measuring the distance traveled in the top zone (Figure 2B), time in top/bottom zone (Figure 2C), several entries in the top zone (Figure 2D), freezing (Figure 2E), average velocity (Figure 2F), and total distance traveled (Figure 2G). In Figure 2B, zebrafish with high anxiety would travel more distance in the bottom of the tank. The group that received the HE administered at a dose of 1 mg/L showed the greatest distance traveled by fish in the upper area compared to the SCOP group (*p* < 0.0001). ANOVA analysis identified these results: F(5,54) = 14.30, *p* < 0.0001. Simultaneously, in the NTT task, the one-way ANOVA revealed a significant effect of the treatment on the time spent in the top/bottom zone F(5,108) = 47.03, *p* < 0.0001 (Figure 2C), the number of entries in the top zone F(5,54) = 7.632, *p* < 0.0001 (Figure 2D), freezing F(5,54) = 12.54, *p* < 0.0001 (Figure 2E), average velocity F(5,54) = 1.80, *p* < 0.0001 (Figure 2F), and on the total distance traveled F(5,54) = 8, *p* < 0.0001 (Figure 2G). The total duration of all freezing bouts indicates increased anxiety and is higher in stressed zebrafish (Figure 2E). Freezing behavior displayed in zebrafish in the novel tank test can be used as an indication of anxiety-like behavior induced by treatment. In Figure 2E, the total duration of all frost episodes indicates increased anxiety and is generally longer in stressed zebrafish. In this study, the group with SCOP (100 µM) showed significant differences compared to the control group (*p* < 0.0001), which had fewer episodes of stress. The freezing behavior displayed in zebrafish in the new tank test can be used as an indication of treatment-like anxiety-like behavior. Strong effects in this evaluation had HE administered at a dose of 3 mg/L. The results are statistically significant. Freezing episodes in the group receiving the 3 mg/L doses are low, close to those receiving imipramine (IMP) (*p* < 0.0001). This highlights the strong anxiolytic and anti-stress effect of HE given to zebrafish. In Figure 2F, the results are statistically insignificant. ANOVA analysis identified F(5,54) = 1.80, *p* <0.0001. Figure 2G, which shows the total distance traveled by zebrafish from different groups, shows a statistically significant difference (*p* < 0.0001) between SCOP (100 µM) and IMP (20 mg/L). IMP was used as a positive control in the NTT test. The active ingredient imipramine is an antidepressant and belongs to tricyclic antidepressants that increase the concentration of serotonin and norepinephrine [59]. It was the first antidepressant to function reliably and served as a precursor to many other antidepressant agents [59]. The total distance traveled by the zebrafish inside the new tank reflects the general/motor neurological phenotypes. Zebrafish is sensitive to nonspecific motor deficiencies and sedative effects of drugs such as SCOP [60,61], but IMP given at a dose of 20 mg/L has significantly stopped these effects. The HE greatly prevented the hypolocomotion and memory deficits caused by the SCOP administration, as evidenced by doses of 1 mg/L and 3 mg/L. The anxiolytic-like effect of the HE treatment was noticed by decreasing the time spent in the bottom zone of the tank (*p* < 0.0001) (Figure 2B) as compared with the SCOP-alone treated animals. Our experimental data indicate that the HE has anxiolytic activity and exerts neuroprotective effects against oxidative stress-induced by SCOP. Anticholinergic drugs, like SCOP, can disrupt short-term or working memory in humans and animals by blocking muscarinic receptors in these brain regions [61]. Over the last decade, zebrafish has become valuable as a complementary model in behavioral pharmacology, opening a new avenue for understanding the relationships between drug action and behavior. Zebrafish offer great advantages of the economy compared to their rodent counterparts; their complex brains and behavioral repertoire offer great translational potential relative to in vitro models [32]. The development and validation of various tests to measure behavior, including cognition, in zebrafish have set the stage for using this animal for behavioral pharmacology studies. Zebrafish was useful in behavioral toxicology and genetics and is an excellent model for neurobehavioral disorders [30,41]. In this study, we presented zebrafish in behavioral pharmacology studies investigating cognition (including the effects of SCOP and HE).

### 3.2. The Effects of H. erinaceus Extracts on Cognition in Y-Maze Task

**Figure 3:**
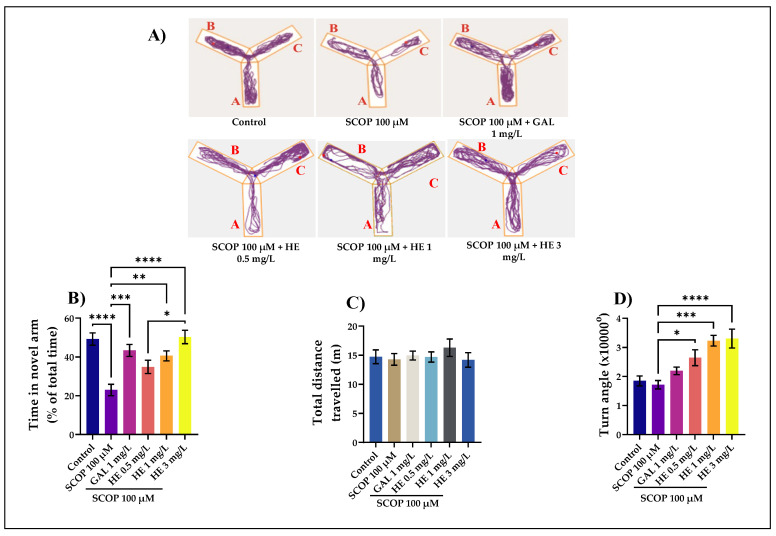
*Hericium erinaceus* ethanolic extract (HE: 0.5, 1, and 3 mg/L) improved the locomotion pattern and memory in the Y-maze test. (**A**) Representative locomotion-tracking pattern of the control, scopolamine (SCOP: 100 µM), *Hericium erinaceus* (HE: 0.5, 1, and 3 mg/L) and galantamine (GAL: 1 mg/L) treated groups. (**B**) Representation of the total time spent in the novel arm in different groups. (**C**) Representation of the total distance traveled by zebrafish in the tank in different groups. (**D**) Representation of the turn angle of zebrafish in the tank in different groups. Values are means ± S.E.M. (n = 10). For Tukey’s *post hoc* analyses: **** *p* < 0.0001, *** *p* < 0.001, ** *p* < 0.01, and * *p* < 0.05.

The Y-Maze memory challenge has the benefit of being easy and quick to learn [37]. The typical locomotion-tracking pattern (Figure 3A) illustrates the differences in swimming traces among the Y-maze arms. Exposure to HE in concentrations of 1 mg/L (*p* < 0.01) and 3 mg/L (*p* < 0.0001) induced, in laboratory animals with cognitive impairment, a substantial increase in the time spent in the new arm. At the dose of 3 mg/L, these results are statistically significant compared to those that received SCOP. Regarding the total distance traveled by zebrafish, the results are not significant. Zebrafish having approximately the same distance traveled without causing significant intensification of locomotor activity. For ANOVA analysis, the results are F(5,54) = 0.45, *p* <0.80. The greater amount of time spent in the novel arm when administering HE (3 mg/L) is an indicator of successful working spatial memory. The zebrafish had higher exploratory behavior to the novel arm, demonstrating that these fish have good learning in memorizing the visual cue arms. SCOP-exposed zebrafish explored the novel arm less, suggesting deficits in response to novelty. Moreover, some endpoint behaviors were measured during the test. The turn angle (Figure 3D) represents the sum of all vectors’ angle of movements created from one position to the animal’s center point to the next. In Figure 3D, which represents the turn angle of zebrafish in the tank, the values are as follows for ANOVA analysis: F(5,54) = 10.02, *p* < 0.0001. The administration of SCOP affects locomotion, as evidenced by the decreasing of turn angle (Figure 3D) compared to HE with 1 mg/L (*p* < 0.001) and 3 mg/L (*p* < 0.0001). Additionally, both doses of HE, but especially the one of 3 mg/L, also increased the absolute turn angle of the fish, thus supporting an enhancing effect of the treatment on the locomotor behavior of the zebrafish. This shows us positive results in zebrafish that received HE reflected by regular movements and increased mobility. Locomotion of the fish was broken up by small movements, changes in direction and this is properly reflected in turn angle. The present data suggest that absolute turn angle is a sensitive measure of motor coordination, since it was affected by drug treatments at doses that failed to alter locomotion [62]. In addition, this shows us the positive response to zebrafish that received HE, highlighted by a good response to novelty and stable spatial memory. All the advantages of the Y-maze memory task and the characterization of neurotransmitter systems related to memory processes in zebrafish indicate that this small teleost can be a good animal model for studying learning and memory [30]. The extract obtained from *H. erinaceus* mycelium was administered intraperitoneally at doses of 10, and 30 mg/kg in APP/PS1 transgenic mice pretreated with amyloid beta for seven days, significantly stimulated memory processes, suggesting that this extract could be beneficial in patients with memory deficits [63]. These results favor the administration of the HE, both in terms of reducing stress for experimental animals and efficacy as an anxiolytic agent. However, there is still a need to test different doses for this protocol and verify this extract’s longer-term administration. These results are like other studies reported by other authors who tested the HE on rats and mice [18,22,23,24,25,26].

### 3.3. The Effects of H. erinaceus Extracts on Recognition Memory in NOR Test

**Figure 4:**
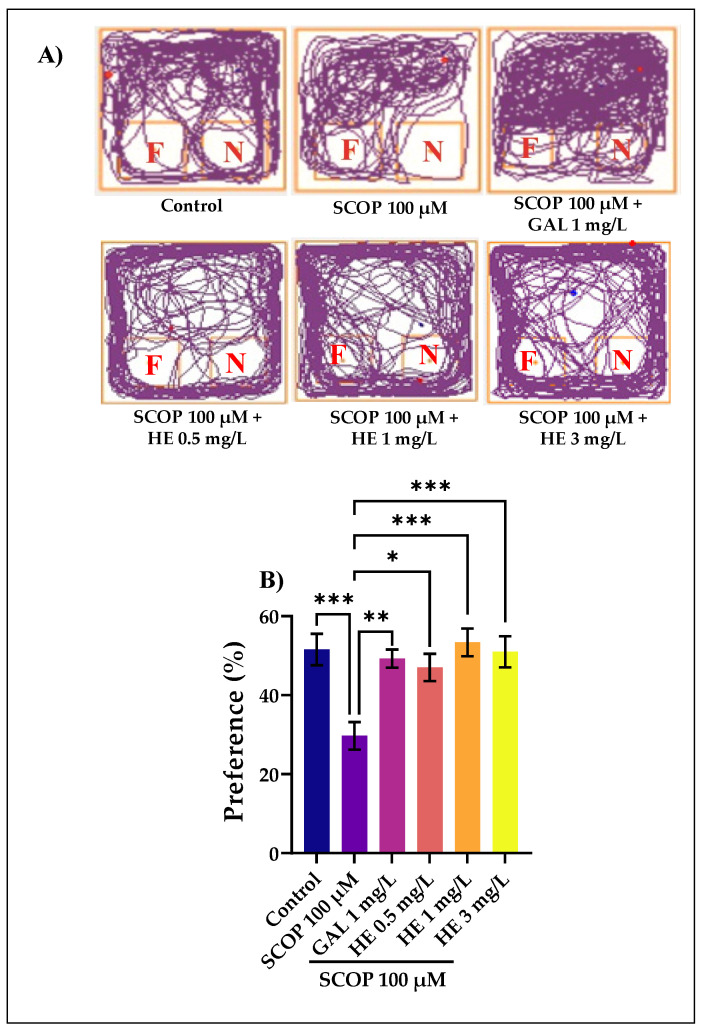
*Hericium erinaceus* ethanolic extract (HE: 0.5, 1, and 3 mg/L) improved memory in the novel object recognition test (NOR). (**A**) Representative locomotion-tracking pattern of the control, scopolamine (SCOP: 100 µM), (HE: 0.5, 1, and 3 mg/L), and galantamine (GAL: 1 mg/L) treated groups. (**B**) Representation of the percentages of preference in different groups. Values are means ± S.E.M. (*n* = 10). For Dunn’s post hoc analyses: *** *p* < 0.001, ** *p* < 0.01, and * *p* < 0.05.

In this experiment, we used object discrimination tasks to evaluate the cognitive effects of the HE in a zebrafish model of cognitive impairment induced by immersion in SCOP. The typical locomotion tracking pattern (Figure 4A) illustrates the differences in the exploration of the familiar object (F) and the novel object (N) within the NOR. It shows that the SCOP-treated group exhibited a high preference to explore F, indicating memory deficits. In the NOR test, one-way Kruskal-Wallis test revealed a significant effect of treatment on preference percentages H(6) = 14.17, *p* < 0.001. GAL used as a positive reference drug evoked memory-enhancing effects, as noticed by the behavioral parameters, supporting the data delivered by the Y-maze test. The preference percentages have been determined as (time of exploration of N/time of exploration of F + time of exploration of N × 100). Animals treated with SCOP showed fewer percentages of preference (*p* < 0.001) (Figure 4B) compared with the control group. In contrast, the administration of HE in the SCOP-treated fish improved the percentage of preferences for N (*p* < 0.0001 for the 1 and 3 mg/L doses), suggesting a memory-enhancing profile. Improving the memory in this zebrafish test is most strongly observed when the HE was administered in high doses (1 mg/L and 3 mg/L). In addition, one-way Kruskal-Wallis test identified overall significant differences between the experimental groups for (B) H(6) = 13.55, *p* < 0.0001. The NOR test provided valuable insight into the cognitive abilities of fish and demonstrated its essential value and contribution in cross-species comparisons. Like rats and mice, zebrafish, guppies, and other fish could discriminate between a novel and a familiar object [64]. Zebrafish could also perform an episodic-like memory test. The NOR task has become a widely used model for the investigation into memory alterations. The preference for a novel object means that the presentation of the familiar object exists in animals’ memory [65]. In comparison to rodents, zebrafish tend to be more appealing choice due to their high reproductive rate, fast growth rate, and low husbandry requirements [39].

### 3.4. Biochemical Parameters Assay in the Brain Tissue

**Figure 5:**
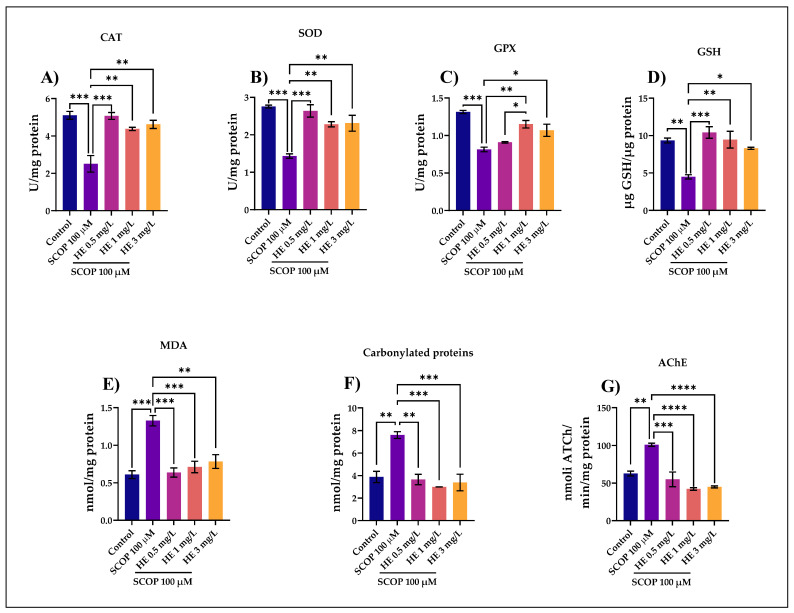
*Hericium erinaceus* ethanolic extract (HE: 0.5, 1, and 3 mg/L) exhibited an anti-AChE effect and improved the antioxidant status in the zebrafish brain. (**A**–**D**) Representation of the enzymes specific activity (CAT, SOD, GPX and GSH) in different groups; (**E**,**F**) Representation of the MDA and protein carbonyl levels in different groups; (**G**) AChE-specific activity. Values are means ± S.E.M. (*n* = 10). For Tukey’s post hoc analyses: **** *p* < 0.0001, *** *p* < 0.001, ** *p* < 0.01, and * *p* < 0.05.

Figure 5A shows the statistical results for the CAT assessment. This enzyme is one of the most abundant enzymatic antioxidants that reduce the levels of reactive oxygen species; studies in small laboratory animals indicate that the activity of this enzyme decreases with age [66]. For this part, ANOVA analysis identified these results: F (4,45) = 17.20. This study showed that the HE in doses of 0.5, 1, and 3 mg/L could modulate CAT activity in the brain tissue. We observed that the administration of the HE in the low dose of 0.5 mg/L determined statistically significant increases in the brain CAT activity, the statistical significance was obtained by reference to the enzymatic activity of CAT specific to the group treated with SCOP (*p* < 0.001). Compared to the group treated with SCOP, it was observed that CAT activity was also intensified by treatment with the HE in doses of 1 and 3 mg/L (*p* < 0.01), but not as significant as the extract administered in the low dose.

In Figure 5B, for the specific activity of the antioxidant SOD estimated in brain homogenates in zebrafish, the one-way ANOVA test showed the existence of significant differences between groups of animals F(4,45) = 16.28, *p* = 0.0002. As shown in the SOD activity, the results are similar to those obtained after evaluating the CAT enzyme. Specifically, the low-dose of the HE (0.5 mg/mL) had a more significant influence on SOD activity compared to the group receiving SCOP (*p* < 0.001). However, the extracts administered in doses of 1 mg/L and 3 mg/L also had significant effects, compared to the group used to control SCOP (*p* < 0.01). SOD is a key cellular antioxidant and is considered an anti-aging enzyme, with studies showing that a 75% reduction in SOD activity causes an accelerated aging process [67]. Over time, animal studies have shown a correlation between aging and decreased SOD activity [48]. In this study, it was observed that in the brain tissue, SOD activity was influenced by the HE in doses of 0.5 mg/L, 1 mg/L, and 3 mg/L comparing to the control group and the group treated with SCOP.

In Figure 5C, for the specific activity of the GPX enzyme, the one-way ANOVA test showed significant differences between groups of animals F (4,45) = 18.34, *p* = 0.0001. GPX is one of the enzymes that provide the primary antioxidant defense against reactive oxygen species, along with catalase and superoxide dismutase [68]. This study showed that HE induced higher GPX activity in the group exposed to a dose of 1 mg/L compared to the group with SCOP (*p* < 0.01). In addition, from the comparison between the quantity of the HE (0.5 mg/L) vs. HE (1 mg/L). Here, the value of the coefficient *p* was 0.05. In this study, the extract with the highest increase in GPX activity was at a dose of 0.5 mg/L.

In Figure 5D, for the specific activity of the antioxidant GSH estimated in the brain homogenates in zebrafish, the one-way ANOVA test showed the following value between groups of animals F (4,45) = 12.92, *p* = 0.0006. GSH plays a central role in antioxidant defense, with irreversible cellular damage when cells lose their ability to maintain intracellular glutathione concentrations [52]. Human and laboratory animal clinical studies suggest a decrease in GSH levels with aging, with the oxidation of GSH increasing its oxidized form and a reduction in its reduced state [69]. The rate of GSH synthesis is primarily controlled by the degree of expression and catalytic activity of the enzyme γ-glutamyl-cysteine synthetase and the cellular availability of cysteine [69]. At a doses of 0.5 mg/L and 1 mg/L, HE induced the highest concentrations of GSH, compared to the group treated with SCOP according to Tukey’s post hoc analysis (*p* < 0.001 and *p* < 0.01, respectively). It is observed that in the case of GSH activity, the most potent effect was due to the HE administered in a lower dose. This was also evident in the case of previously evaluated enzymes. GSH was involved in many metabolic processes, effectively eliminating free radicals and other reactive oxygen species (hydroxyl radicals, lipid peroxides, nitrite peroxides, and H_2_O_2_) directly and indirectly through enzymatic reactions [68].

In Figure 5E, for the MDA levels, the one-way ANOVA test showed the following value between the groups of animals F (4,45) = 17.10, *p* = 0.0002. MDA can be found in many biological samples (serum, plasma, urine, tissues, and even food) and has become one of the most widely used indicators to estimate the effects of oxidative stress on lipids [70]. MDA is a recognized marker in the laboratory diagnosis for evaluating oxidative stress, its plasma concentration being an indicator of the degree of lipid peroxidation, a process that depends on the level of free radicals [71]. Our evaluation shows that the HE administered in doses of 0.5 mg/L and 1 mg/L against SCOP again shows the most significant values of the p coefficient (*p* < 0.001). There was a high efficiency in HE administered in a higher dose, of 3 mg/L (*p* < 0.01), but not as significant as the extract administered in lower doses. This again indicates that HE administered in lower quantities has a more substantial role in the antioxidant activity observed in zebrafish.

In Figure 5F, for the carbonylated proteins levels, the one-way ANOVA test showed the following value between groups of animals F(4,45) = 15.92, *p* = 0.0002. Elevated levels of carbonylated proteins, lipid peroxidation leading to oxidative stress may also be a reason for memory loss through the administration of scopolamine [60]. This is also observed in our evaluation because the group of fish that received the dose of SCOP 100 µM showed a high level of carbonylated proteins compared to the control group (*p* < 0.01). Simultaneously, we observe the effect of the administration of the HE in the case of zebrafish with cognitive impairment induced by SCOP. The effect is evident in groups of animals given SCOP and HE at a dose of 1 mg/L and 3 mg/L (*p* < 0.001). Furthermore, good effects were observed at a dose lower than 0.5 mg/L. Considering the results, we observe that SCOP-induced brain oxidative stress is highlighted by increasing carbonylated proteins levels and being a pro-oxidant agent. This was also observed in the case of MDA; the values were closely related to the ANOVA analysis: F(4,10) = 17.10, *p* = 0.0002; F(4,10) = 15.92, *p* = 0.0002. Simultaneously, the administration of HE in high doses can mediate this process of decreasing the level of carbonylated proteins produced by SCOP. Recent studies have shown that serum levels of oxidative stress markers (MDA, carbonylated proteins, etc.) correlate with the clinical severity of AD, being used as prognostic indices for this comorbidity [72].

In Figure 5G, for the specific activity of AChE estimated in the brain homogenates in zebrafish, the one-way ANOVA test showed the existence of significant differences between groups of animals F (4,45) = 24.76, *p* < 0.0001. Our experimental data showed an increase in AChE activity following SCOP administration (*p* < 0.01) compared to the control group. The administration of the HE in high doses of 1 mg/L and 3 mg/L resulted in a significant decrease (*p* < 0.0001) in the specific activity of AChE in a dose-dependent manner compared to the group treated with SCOP. Because AChE inhibitors have potential in animal models of amnesia, attenuation of the brain AChE activity suggests that the HE confers anti-amnesic effects in mice treated with SCOP [25]. AChE is the enzyme that causes acetylcholine to be rapidly hydrolyzed in the synaptic cleft [73]. It is present in the synaptic cleft, near the postsynaptic membrane, where the concentration of acetylcholinesterase is closely interdependent with the activity of the nerve [74,75]. According to the cholinergic hypothesis, inhibition of AChE activity improves cholinergic function in patients with AD [72]. The inhibition of AChE is one of the therapeutic strategies for dementia [76].

### 3.5. Pearson Correlations between Behavioral and Biochemical Parameters

**Figure 6:**
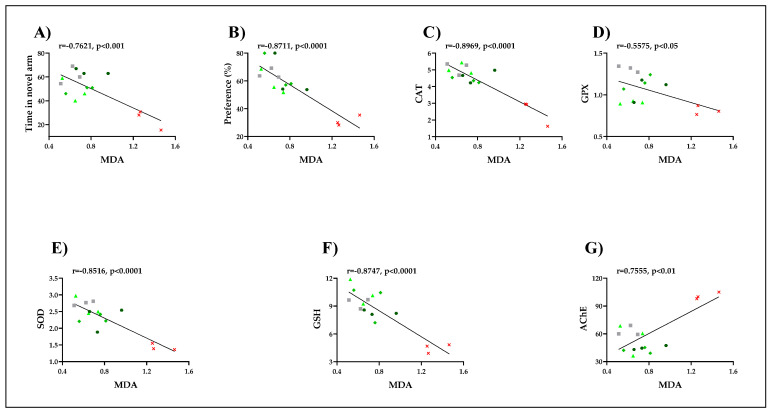
Correlation analyses between behavioral and biochemical parameters (Pearson’s correlation, *n* = 10): (**A**) Time in novel arm (% of total time) vs. MDA: *r* = −0.7621, *p* < 0.001; (**B**) Preference (%) vs. MDA: *r* = −0.8711, *p* < 0.0001; (**C**) CAT vs. MDA: *r* = 0.8969, *p* < 0.0001; (**D**) GPX vs. MDA: *r* = −0.5575, *p* < 0.05; (**E**) SOD vs. MDA: *r* = −0.8516, *p* < 0.0001; (**F**) GSH vs. MDA: *r* = −0.8747, *p* < 0.0001; (**G**) AChE vs. MDA: *r* = −0.7555, *p* < 0.01. Data expressed are time in the novel arm (% of total time), time in the novel arm (s), SOD (U/mg protein), CAT (U/mg protein), GPX (U/mg protein), GSH (µg GSH/µg protein), AChE (nmol/min/mg protein), and MDA (nmol/mg protein).

In this study, the Pearson correlation coefficient (*r*) quantifies the linear association between different behavioral and biochemical parameters. Our results revealed that the representation of the total time spent in the novel arm in different groups (*r* = −0.7621, *p* < 0.001) measured in the Y-maze task and the representation of the percentages of preference in different groups (*r* = −0.8711, *p* < 0.001) determined in a novel object recognition task strongly correlate with MDA, the product of lipid peroxidation. This suggests that the memory improvement of the zebrafish by the HE is well correlated with a decrease in the brain MDA levels. Moreover, we also correlated AChE and several defense systems, including CAT, GPX, SOD, and GSH, with MDA. In Figure 6G, which represents the correlation between AChE and MDA, a positive correlation was observed in which *r* = 0.7555 and *p* < 0.01. This suggests that the reduction in the specific activity of AChE is well correlated with a low level of lipid peroxidation. More importantly, strong negative correlations were observed between all specific enzymes analyzed and MDA level regarding the antioxidant system. In Figure 6C, which represents the correlation between CAT and MDA, a negative correlation was observed in which the value of *r* = −0.8969 and *p* < 0.0001. Additionally, in Figure 6D, when linear regression was determined, significant correlations between GPX and MDA were evidenced. A negative correlation was observed between *r* = −0.5575 and *p* < 0.05. Likewise, in Figure 6E, which represents the correlation between SOD and MDA, a negative correlation was observed between *r* = −0.8516 and *p* < 0.0001. In addition, in Figure 6F, which represents the correlation between GSH and MDA, a negative correlation was observed between *r* = −0.8747 and *p* < 0.0001. SCOP significantly increases AChE and MDA levels in the brain. Our results indicated that SCOP increased brain MDA levels. The HE decreased brain MDA levels in experimental groups. MDA is one of the major aldehydes formed after the breakdown of lipid hydroperoxides [70]. Therefore, it is considered a good biomarker of free radical damage in pathologies associated with oxidative stress [77]. MDA is also a toxic compound in mutagenesis in the elderly, carcinogenesis, diabetic nephropathy, radiation-induced lesions, and several other pathological processes [78]. Numerous studies have confirmed the close link between excessive free radical formation and degenerative diseases such as atherosclerosis or AD [79]. Thus, in the brains of deceased patients who have AD were found significantly higher concentrations of MDA than the concentration found in the brains of healthy people of the same age [80]. According to the results presented, the administration of the HE in animals pretreated with SCOP resulted in a significant increase in GSH simultaneously with a significant decrease in the level of carbonylated proteins and MDA in a dose-dependent manner compared to the group treated with SCOP. Numerous experimental studies have reported a correlation between SCOP-induced memory degradation in zebrafish and oxidative stress in amnesic patients [60,61]. Many other authors have reported the neuroprotective effect of the *H. erinaceus* mushroom in animal studies [16,18,20,23,25,63,81]. Coherent with these studies, we found that *H. erinaceus* sustained memory formation and exhibited an antioxidant profile along with a decrease in brain AChE activity.

## 4. Discussions

Through the data obtained following the investigation of the neuroprotective action of the HE, the pharmacodynamic effects of this pharmacologically active extract can be highlighted. The HE reduce the symptoms of memory loss and prevent neuronal damage caused by beta-amyloid plaques, which accumulate in the brain during AD [26]. The HE have also improved cognitive function and cholinergic brain function by protecting neurons from SCOP-induced neurotoxicity [20]. Together, these data support the hypothesis that the HE may modulate memory processing, suggesting that these compounds present a potential preventive strategy against cognitive impairment. *H. erinaceus* improves brain function and may have beneficial applications in relieving the symptoms of AD [63]. There was considerable evidence that SCOP causes oxidative stress through the interference with acetylcholine in the brain leading to cognitive impairment [60]. In this study, we investigated whether the HE could inhibit the memory impairment induced by SCOP by inhibiting AChE or decreasing oxidative stress. The restored degree of impairment was gauged using both NTT, NOR, and the Y maze tests with or without treatment. AChE inhibitors, which enhance the availability of acetylcholine in the synaptic cleft, could reverse the SCOP-induced deficit [82]. The novel AChE inhibitors from mushroom sources could be valuable alternatives in the context of AD treatment. Several studies have shown the cognition-enhancing properties of natural products and their components using different animal models [60,82]. Biochemical parameters, indicators of oxidative stress were determined using cellular homogenates from laboratory animals with SCOP-induced cognitive impairment and treated with the HE administered in concentrations of 0.5 mg/mL, 1 mg/mL, and 3 mg/mL to evaluate the potential of these solutions to counteract the effects induced by reactive oxygen species. Following this study, the following aspects were highlighted: MDA and carbonylated protein levels were affected by the administration of the HE in laboratory animals with cognitive impairment. The administered extract induced statistically significant changes in the brain in all three concentrations. Significant increases in total reduced GSH content were induced by the HE. Additionally, the enzymatic activity of CAT was modulated by the administration of the HE. Moreover, SOD activity was intensified by the administration of the HE. Data from the literature have shown that decreased levels of acetylcholine in the hippocampus due to increased activity of AChE could degrade the movement of the cholinergic system in the brain, causing cognitive dysfunction [82,83]. Our experimental results from the Pearson correlation indicate that the stimulation of memory processes assessed by specific behavioral tests is correlated with decreased oxidative stress and AChE activity in the brain of SCOP pretreated animals receiving the HE.

## 5. Conclusions

It can be stated that the lyophilized extract of *H. erinaceus* obtained using an ultrasonic extraction method and enriched in erinacine A represents an efficient bioformulation for testing to monitor the role of neuroprotective effects. This is the first study investigating the antioxidant and neuroprotective potential of *H. erinaceus* fungal biomass extract obtained from the ultrasonic extraction method. These results indicate the medicinal and antioxidant properties of *H. erinaceus* in alternative medicine for cognitive impairment, and *H. erinaceus* compounds could be used for treating various neurodegenerative diseases as antioxidative functional ingredients. The HE could repair effects on memory and behavioral disorders produced by SCOP and may have beneficial effects in AD treatment.

## Data Availability

Data is contained within the article.
